# Priority interventions to reduce HIV transmission in sex work settings in sub-Saharan Africa and delivery of these services

**DOI:** 10.7448/IAS.16.1.17980

**Published:** 2013-03-04

**Authors:** Matthew F Chersich, Stanley Luchters, Innocent Ntaganira, Antonio Gerbase, Ying-Ru Lo, Fiona Scorgie, Richard Steen

**Affiliations:** 1 Centre for Health Policy, School of Public Health, Faculty of Health Sciences, University of the Witwatersrand, Johannesburg, South Africa; 2 International Centre for Reproductive Health, Department of Obstetrics and Gynaecology, Ghent University, Belgium; 3 Centre for International Health, Burnet Institute, Melbourne, Australia; 4 World Health Organisation Regional Office for Africa, Brazzaville, Democratic Republic of Congo; 5 Department of HIV/AIDS, World Health Organization, Geneva, Switzerland; 6 Maternal, Adolescent and Child Health (MatCH), Department of Obstetrics and Gynaecology, Faculty of Health Sciences, University of the Witwatersrand, Johannesburg, South Africa; 7 Department of Public Health, Erasmus MC University Medical Centre, Rotterdam, the Netherlands

**Keywords:** sex work, prostitution, HIV, sub-Saharan Africa, HIV prevention, systematic review, condoms

## Abstract

**Introduction:**

Virtually no African country provides HIV prevention services in sex work settings with an adequate scale and intensity. Uncertainty remains about the optimal set of interventions and mode of delivery.

**Methods:**

We systematically reviewed studies reporting interventions for reducing HIV transmission among female sex workers in sub-Saharan Africa between January 2000 and July 2011. Medline (PubMed) and non-indexed journals were searched for studies with quantitative study outcomes.

**Results:**

We located 26 studies, including seven randomized trials. Evidence supports implementation of the following interventions to reduce unprotected sex among female sex workers: peer-mediated condom promotion, risk-reduction counselling and skills-building for safer sex. One study found that interventions to counter hazardous alcohol-use lowered unprotected sex. Data also show effectiveness of screening for sexually transmitted infections (STIs) and syndromic STI treatment, but experience with periodic presumptive treatment is limited. HIV testing and counselling is essential for facilitating sex workers’ access to care and antiretroviral treatment (ART), but testing models for sex workers and indeed for ART access are little studied, as are structural interventions, which create conditions conducive for risk reduction. With the exception of Senegal, persistent criminalization of sex work across Africa reduces sex workers’ control over working conditions and impedes their access to health services. It also obstructs health-service provision and legal protection.

**Conclusions:**

There is sufficient evidence of effectiveness of targeted interventions with female sex workers in Africa to inform delivery of services for this population. With improved planning and political will, services – including peer interventions, condom promotion and STI screening – would act at multiple levels to reduce HIV exposure and transmission efficiency among sex workers. Initiatives are required to enhance access to HIV testing and ART for sex workers, using current CD4 thresholds, or possibly earlier for prevention. Services implemented at sufficient scale and intensity also serve as a platform for subsequent community mobilization and sex worker empowerment, and alleviate a major source of incident infection sustaining even generalized HIV epidemics. Ultimately, structural and legal changes that align public health and human rights are needed to ensure that sex workers on the continent are adequately protected from HIV.

## Introduction

Sex work is common in sub-Saharan Africa where an estimated 0.7 to 4.3% of women exchange sex for money, goods or favours [1]. These women carry a markedly high burden of HIV (almost 13-fold greater than women in the general population) and other sexually transmitted infections (STIs) [2]. Much of HIV risk for these women is a manifestation of their extraordinary social and economic vulnerability, and the high levels of stigma and violence attached to sex work [3].

High rates of partner change mean that HIV exposure and onward transmission are frequent. Though the exact extent of the contribution is contested, clearly sex workers, and their clients and boyfriends, play an important role in early and advanced HIV epidemics in sub-Saharan Africa [4,5]. Yet, only about one in three female sex workers (FSWs) in the region receive adequate HIV prevention services [6], and even fewer access HIV treatment, care and support [7]. Most interventions addressing sex workers and clients in Africa operate in isolation with little or no support from national governments or international donors and consequently have limited coverage [8].

A modelling study indicated that raising condom use in sex work settings along transport routes in East Africa from the present 78 to 90% would avert two-thirds of incident HIV infections [9]. Programmes in several other settings have shown that a few interventions, implemented at sufficient scale, can markedly reduce the burden of HIV among sex workers and their clients and make considerable population-level impact [10–12]. This systematic review examines evidence of the effectiveness of interventions to reduce HIV transmission among FSWs in sub-Saharan Africa. The review therefore identifies priority HIV prevention interventions, discusses approaches to delivery and highlights important knowledge gaps.

## Methods

Data were collated from research studies among FSWs in sub-Saharan Africa. The Medline (PubMed) database was searched for relevant English articles published between January 2000 and July 2011. Search terms were: prosti (or any term with this word) or “sex work” or “sex worker” or “sex workers”, and Africa (MeSH term or any field). A key non-indexed journal *Research for Sex Work* (http://www.researchforsexwork.org) and references of review articles and studies located in the review were hand searched. We included findings of other systematic reviews on pertinent topics, for example, condom promotion [13]. Web sites of agencies involved in HIV prevention were searched (UNAIDS, Family Health International and Population Council). Experts in the field provided additional articles and some grey research reports.

To be eligible for inclusion, articles had to contain information on outcomes of an intervention to reduce transmission or acquisition of HIV or other STIs among FSWs in sub-Saharan Africa. Publications were only included if they reported at least one outcome measure that could be externally validated. This included biological outcomes (prevalence or incidence of HIV or other STIs) or measurable health outcomes (condom use, number of sexual partners, health-service utilization, HIV testing uptake, antiretroviral treatment (ART) initiation and HIV-related knowledge). Included were experimental (randomized controlled trials), cohort, repeat cross-sectional studies, or cross-sectional studies that reported associations between exposure to an intervention and a relevant outcome. Study participants were limited to FSWs, defined as women who exchange sex for money or other gifts and commodities. Studies among groups at high-risk, such as female bar workers, were excluded.

**Figure 1:**
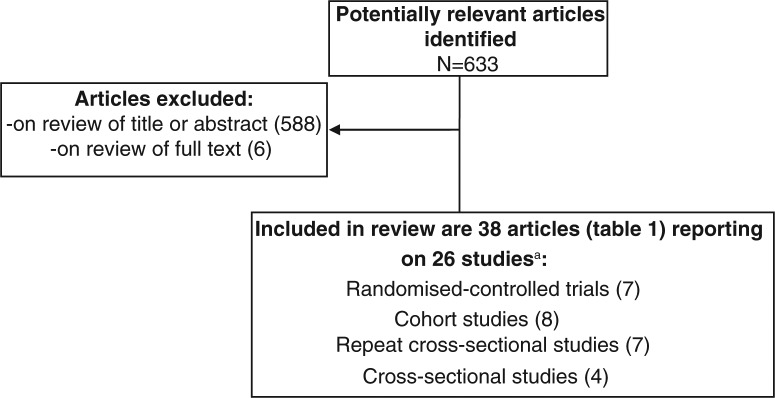
Flow chart of identification and selection of studies.

^a^Results from some studies were published in several articles.

The Medline search identified 633 articles (Figure 1), which were imported into Endnote. Twenty six studies described in 38 articles were identified after a single reviewer screened the article titles, abstracts and then full text of articles. A single reviewer extracted data about the sex work setting, study population, intervention description, study methods, outcomes listed above and study limitations (Table S1).

Interventions were classified according to a conceptual framework, adapted from a WHO framework which depicts complementary approaches to HIV prevention in sex work settings [14]. Specifically, interventions for reducing HIV transmission in sex work settings are classified into three broad categories: 1) limiting exposure to HIV and other STIs; 2) reducing HIV transmission efficiency; and 3) addressing sex worker empowerment and structural conditions (the physical and social environment in which individual behaviour takes place) [15]. Considerable heterogeneity of interventions, study populations and outcomes measured precluded use of meta-analysis estimates of overall measures of effect [16]. Instead, findings are summarized in results text, which also explores causes of heterogeneity of outcomes between studies. Results are presented in Table S1, ordered by rigour of study design and region of Africa (West, Central east and Southern regions).

## Results

### Limiting exposure to HIV and other STIs: reducing unprotected sex

Promotion of condoms and their availability free or at low cost in clinics or through outreach are interventions common to nearly all effective sex work projects [8]. Both community mobilization through peer workers and structural interventions (such as micro-enterprise support [17]) appear to make a critical contribution to increasing condom use in sex work (Table S1).

Few studies provided data on whether increased condom use impacted on HIV rates [18–21]. Though HIV levels reduced substantially and condom levels rose in a repeat cross-sectional study in Côte d'Ivoire, the authors suggest caution in interpreting their findings given the considerable changes in composition of the sex worker population [20]. Most studies assessed the effectiveness of combination-intervention packages, rather than condom promotion or provision alone, and several observational cohorts link increasing condom use with declining STI incidence or prevalence among FSWs and their clients. This includes studies in countries as diverse as Benin [18], the Congo [22], Côte d'Ivoire [20], Kenya [23] and South Africa [24].

Studies in Malawi, Kenya and South Africa found high levels of acceptability and utilization of the female condom [25–27], but lesser effects in Zimbabwe [28]. Within a service already providing male condoms, the addition of female condoms increased overall levels of condom use, but some replacement of male condoms with female condoms also occurred [25].

Several studies, but not all [29], suggested that peer-mediated condom promotion is particularly effective in supporting consistent condom use [19–21,25,30–33]. In addition to promotion and distribution, peers should ascertain that FSWs and clients know how to use condoms correctly [34]. Condom promotion by outreach health workers also appears effective [18,35]. Raised condom use and declines in high-risk behaviours were also found in a Kenyan sex worker cohort that received a combination of peer and clinic-based risk-reduction counselling [36]. Further, adding micro-enterprise services to a peer-mediated intervention in Kenya's urban slums (see structural conditions described below) enabled two-thirds of participants to establish businesses and reduced HIV exposure (lower partner number and higher condom use) [17].

There are limited data on whether HIV testing affects sexual behaviour among sex workers. In a Kenyan cohort, compared to HIV-negative sex workers, women who acquired HIV infection did report fewer sexual partners and higher condom use subsequent to HIV testing [37]. In a study in Senegal, having had an HIV test was not associated with condom use with clients (adjusted prevalence ratio=0.98), both among women testing HIV positive and those testing negative. A concerning finding was that sex workers who had had an HIV test reported lower condom use with regular partners than women who had not tested [38].

### Reducing HIV transmission efficiency

Treatment of STIs [39] and of HIV [40] can reduce HIV transmission efficiency. No studies were, however, located that had specifically aimed to improve sex workers’ access to ART. Several projects in Africa, however, have registered success in improving FSWs’ access to STI services, through one of three approaches, or a combination thereof: management of symptomatic STIs, STI screening and periodic presumptive treatment (PPT) for asymptomatic infections [41–43].

A cohort study in Nairobi, Kenya, that included STI screening, found that the per-act rate of HIV acquisition among FSWs declined dramatically between 1985 and 2005 and correlated closely with decreases in the prevalence of gonorrhoea and genital ulcers [44]. These declines predated reductions in HIV prevalence among the general population in Kenya. In Benin, syndromic STI management, based on standardized algorithms, detected just under half of cervical infections when used as a screening tool among sex workers participating in a microbicide study [45]. Nearly 60% of those with an infection did not return for their scheduled visit to obtain the screening test results. The authors postulate that low return may be due to the high mobility of the population; the women, mostly asymptomatic, deciding to avoid the transport costs and time of return visits; or symptomatic women purchasing antibiotics in informal settings instead. Screening for syphilis appears feasible and cost-effective for identifying asymptomatic infection [18,20,24,44,46].

Project Sida-2 in Benin attributed decreases in HIV and STI prevalence in the late 1990s both to increasing condom use with clients and to provision of STI screening services in the sex worker clinic [18]. In other parts of Benin, without targeted interventions, HIV prevalence among sex workers increased substantially over the same period. Similarly, in a study in Côte d'Ivoire, the incidence of HIV fell in all women who received STI screening, whether basic or more intensive [47]. In some parts of Africa, STI screening is provided for registered sex workers attending scheduled clinic visits. Regular screening of sex workers in Senegal, where sex work is decriminalized, has been credited with contributing to a low and stable HIV prevalence in the country [48,49]. Sex workers in Senegal older than 21 years are required to register and attend monthly clinic visits for examination, STI screening, free condoms, family planning counselling and even provision of services for their children [49]. Many sex workers, however, choose to remain unregistered (“*les clandestines*”) [50]. A portion of these were below 21 years, the legal age of sex work, while others lacked the necessary identity papers; were unaware of the registration system or its procedures; were previously registered but had discontinued sex work for a period and not re-registered; were simply rejected the idea of having to register; or were believed the system compromised their discretion.

PPT involves antibiotic treatment of common STIs and is mostly used where the prevalence of curable STIs is high and screening is not feasible [41,43]. Observational [24,51–53] and trial evidence [54–56] supports the effectiveness of this approach, and pathogens covered by PPT regimens in Africa have included *Neisseria gonorrhoeae*, *Chlamydia trachomatis, Haemophilus ducreyi* and *Treponema pallidum*.

Several service delivery models were explicitly designed to reduce stigma for FSWs and optimize the safety and convenience of services. These include use of night clinics [33] or taking STI treatment directly to sex workers and clients in venues such as hotels [57]. Peer educators have also been deployed to raise awareness about STIs, promote STI services, reinforce correct use of STI medications, and facilitate follow-up visits and partner education and treatment [21,58]. A cross-sectional study in Abidjan, Côte d'Ivoire, at 29 health care facilities and 10 pharmacies found that perceptions of low quality or stigmatising attitudes in the public sector meant that many FSWs preferred accessing private providers or pharmacists for STI treatment. These private facilities were often poorly equipped, of low quality and more expensive than public facilities [59].

### Addressing sex worker empowerment

Relatively few studies reviewed included empowerment interventions, and none were identified that sought to alter the broader political economy and transformation in that sphere. In projects that did report outcomes of empowerment interventions, their services with sex workers had initially focused narrowly on a limited number of health sector activities and services. With time, however, these projects involved sex workers in the response, aiming to encourage and facilitate broader empowerment [17,21]. An information and counselling intervention in Sierra Leone increased sex workers’ ability to initiate discussion of condom use with their partners [35]. Information on risk reduction was commonly provided to sex workers when they attended health services, but also through regular outreach, peer education and activities offered through community-level drop-in centres [21]. A trial among 93 women in Pretoria, South Africa, that aimed to empower sex workers by building their skills in negotiating safer sex practices, refusing uncooperative clients and self-protection in violent situations, reported fewer STI symptoms in the intervention group [27]. This intervention applied educational and counselling strategies to equip women with violence prevention skills, such as avoiding hazardous alcohol-use, exiting volatile situations and actively seeking community resources [27]. No evidence was located on the effectiveness of other strategies.

## Discussion

This review of sub-Saharan Africa noted limited progress in the implementation, evaluation and scale-up of sex worker interventions compared to Asia. Though some projects measured changes in occurrence of STIs, studies with HIV incidence as an outcome were surprisingly rare. As with HIV prevention in all populations, no single intervention by itself is sufficiently effective in reducing HIV risk in sex workers. Initial combinations of services have included support for condom promotion and STI control in sex work settings. With the maturity of programmes, increasingly synergistic combinations of services can be implemented, that address the multiple levels at which sex workers experience vulnerability and risk [30,42,60].

Next we draw some conclusions and implications for future programming, based on the data reviewed. Some limitations, however, need to be considered. First, the study applied a strict definition of sex work when selecting eligible articles, an important limitation given that transactional sex is very common in Africa [61–64] and that it is oftentimes difficult to draw definitive distinctions between this and sex work. Further, it is difficult to directly compare finding between studies, as there is considerable variation in the definitions of sex work applied, as well as in the settings in which sex work takes place in Africa.

### Limit exposure

The review did not identify studies assessing the effectiveness of condom promotion or provision alone on HIV incidence. An intervention providing free condoms and STI services to FSWs in Kinshasa increased condom use and reported lower prevalence of HIV and other STIs, despite fewer than 60% of the women reporting condom use with all clients. Client refusal was the main obstacle to 100% condom use [21,22]. Nine studies from Africa were included in a systematic review in 2007, which collated substantial evidence that interventions targeted at FSWs can achieve large increases in condom use. Overall, 15 of 19 studies with FSWs showed increased condom use, which doubled in eight studies [13].

Heavy drinking and drug taking by sex workers or their clients can undermine condom use [65], as alcohol may adversely affect sexual decision making, condom negotiation and their correct use [66]. A trial in South Africa evaluated a combined counselling and education intervention to enhance risk-reduction skills and increase self-efficacy among sex workers who use drugs, and found a decrease in substance use during sex work and fewer STI symptoms in the intervention group than in controls [27]. Despite much evidence of the effectiveness of alcohol-control measures [67], only one intervention to support the adoption of safer drinking patterns was identified. In particular, use of “brief interventions” – which are cost-effective and recommended for wide use – should be investigated in sex work settings [66–68].

Contraceptive choice and increased control over fertility is an important factor influencing sex workers’ economic vulnerability and consequently their options of exiting sex work or declining clients unwilling to use a condom [3,69,70]. Two studies reported evidence about the promotion of condoms for dual protection in sex work settings in Africa [71,72]. An observational study in Addis Ababa, Ethiopia, found higher levels of consistent condom use in sex workers using condoms for contraceptive purposes [72]. However, FSWs in Kenya who said they use condoms for contraception often reported inconsistent condom use, suggesting high contraceptive failure rates are likely [71].

### Transmission efficiency

Despite increases in the availability of HIV testing across sub-Saharan Africa, up to 60% of sex workers remain unaware of their status and thus unable to access ART [7]. Almost half of FSWs in one study only learnt their status during pregnancy [73]. Importantly, there is only limited evidence that HIV testing or “prevention for positives” leads to increased condom use among sex workers [37,38,74]. There is thus uncertainty about whether the effects of HIV testing on safer sexual behaviour noted in the general population, most especially among those testing HIV positive [75,76], extend to sex workers. No sex work studies were included in meta-analyses on this topic. Given that condom use in sex work is strongly influenced by client motivation, sex workers’ scope and incentive for behaviour change following HIV testing may be limited. Also, further study is needed to assess how sex workers perceive HIV testing and to validate HIV testing models in sex work settings. Promotion of testing through peer outreach may be a more acceptable approach [77].

HIV care and ART for HIV-positive sex workers decreases their morbidity and mortality and reduces subsequent HIV transmission. Programmes in sex work settings are challenged to respond to the findings of HPTN 052, which demonstrated marked reductions in sexual transmission of HIV among adults with a CD4 cell count 350 to 550 and in stable serodiscordant couples [40].

The presence of an STI is a major determinant of the efficiency of HIV transmission when condoms are not used or fail. A Tanzanian cohort study provided high-risk women including FSWs with three-monthly STI screening and syndromic case management, together with education, condom distribution and HIV testing [78]. Declines were noted in HIV incidence from 13.9/100 to 5.0/100 woman-years over a period of 30 months, with concomitant reductions in STI prevalence.

Rapid point-of-care screening tests may have considerable additional value in these populations [79]. In a study in Benin, all sex workers were willing to wait for about 20 minutes for results of rapid STI tests, but rates of return for results of non-rapid tests were low [45]. A modelling study using data from a sex work project in Cotonou, Benin, found that rapid point-of-care screening tests for cervical infection due to gonorrhoea and chlamydia may be more cost-effective than a syndromic approach, owing to their higher sensitivity and specificity [80]. Others have assessed alternative approaches, including simplified screening algorithms – based on clinical signs (speculum examination) and simple laboratory tests [81].

Reports of poor rates of return for STI screening results and the asymptomatic nature of most STIs mean that PPT has an important role [41,43]. Modelling data from South Africa, Benin and Laos estimated that 40% PPT coverage over 2 years would reduce HIV incidence among sex workers by 20% [82]. WHO recommends PPT as a component of broader STI services for sex workers [83]. Combination therapy using single-dose regimens, such as azithromycin plus cefixime, minimize drug resistance. Once adequate STI services are established and/or infections are controlled at lower levels, PPT can be tapered or discontinued [83]. While it is critical to simplify testing and interventions, equally importantly, efforts are required to raise demand for services and then to actively follow-up women. Women who do not return for test results or scheduled visits should be actively traced, ideally through a peer worker network, or with cell phone contact or related technology. High rates of follow-up are possible [84] but require concerted efforts of the clinical team, with services designed jointly with sex workers and aligned to their needs, such as having night clinics [33], or outreach within brothels [57].

### Structural interventions and empowerment

Structural interventions – activities that tackle conditions inherent to sex work that aggravate vulnerability and risk – have assisted in creating enabling environments for effective programming and for implementing services at scale in other settings [85] but are notably underused in Africa. Interventions that aim to promote individual behaviour change are necessary but, by themselves, are insufficient. They should be supported by interventions that tackle the economic and socio-cultural factors underpinning and deepening the vulnerability of sex workers to HIV [86]. This is very challenging as these factors include the low status of women, poverty and economic marginalization, population mobility, lack of educational and employment opportunities, and local attitudes to sex and sexuality.

Addressing sex workers’ overall well-being and empowerment is essential for minimizing stigma and enhancing acceptability and uptake of services. The provision of respectful, non-judgemental services for sex workers can also catalyze improved living and working conditions and, beyond that, broader social change. Community-based organizations can contribute to creating enabling environments for prevention [87]. Sex work organizations have been credited with facilitating social cohesion, increasing solidarity and mutual support, reframing sex work as valid work and improving overall working conditions for FSWs [85]. Organizations of sex workers may also promote higher payments for sex and other economic empowerment initiatives, which have been associated with a lowering of HIV risk [88]. Examples of such FSW-led networks and organizations were found in Nigeria [89], Mali [90] and South Africa [91]. The health sector can also play a key role in supporting community empowerment among sex workers. Such efforts represent an extension from and reinforcement of more focused health sector interventions.

Several authors argued that the social and legal status of sex work – criminalized throughout Africa aside from in Senegal – reduces FSWs’ control over their working conditions and presents numerous barriers to accessing health services and legal protection [92,93]. Many sex workers on the continent experience violence on the streets while soliciting clients, on the job and in their personal lives [27,94–96]. A study in Senegal reported how involvement of law enforcement agencies and the judiciary went a long way to eliminating violence towards sex workers [49]. Elsewhere this would likely be challenging, however, since law enforcement may exacerbate the risk of violence against sex workers, with police in many cases themselves perpetrators of harassment and brutality [93,94,97,98]. A code of conduct for intermediaries and clients in formal sex work settings was developed in South Africa, although monitoring and enforcing adherence was reportedly difficult in practice [99].

A microfinance programmes for women, which aim to provide sustainable incomes for women and their households reported promising outcomes [17] Such interventions are clearly important to reduce sex workers’ overall vulnerability to HIV, but a report from three countries in southern Africa suggests these are unlikely to work if framed as strategies to “rescue” sex workers from the industry [94].

### Combination interventions

A two-pronged approach would encompass health sector interventions together with complementary efforts to address the structural conditions of sex work on the continent [10,100]. As in Asian countries [101], in the Dominican Republic combining structural interventions (government policy and venue-based interventions) and sex worker solidarity led to greater improvements in condom use outcomes than solidarity interventions alone [102]. At a minimum, high levels of condom availability in sex work settings are required, conforming to quality standards and ideally distributed with lubricants. This was associated with higher use in several studies reviewed here [103,104].

Services that initially address HIV exposure and transmission efficiency – including peer interventions, condom promotion, and respectful and non-judgemental STI services – thus serve as a platform for subsequent community mobilization, and more comprehensive health and social services can be phased in, such as childcare [24,85,105]. Peer-led services, in particular, lend themselves to being progressively supplemented and strengthened with additional interventions. It is vital to engage sex workers in designing such activities which are cognisant of the social, economic and political context, and adopt a community development ethos, as opposed to a narrow biomedical approach [106]. This process can be facilitated through discussions, surveys of sex workers’ information needs and field-testing of materials [58]. Informational materials should be simple, consistent, non-judgemental, attractive and culturally sensitive. Possibly more effective than printed materials [103], however, are other formats such as peer-led drama, video sessions and role-playing exercises, particularly as oral traditions among sex workers tend to be well developed [20,21,107].

National programmes in Asia have shown that broad coverage of services is possible. With sufficient planning and political will, services to prevent HIV reached three-quarters or more of sex workers across large geographical areas of India [11].

## Conclusions

Public health and human rights are complementary, not conflicting, goals. In sex work, however, public health goals and existing, oppressive laws are often at odds with each other. To date, in no setting has the criminalization of private sex acts between two consenting adults stopped or slowed the spread of HIV. From a public health perspective, criminalization of sex work marginalizes sex workers, which, in turn, constrains efforts to reach the population with needed health services. Ultimately, structural and legal changes that align public health and human rights are needed to ensure that sex workers are adequately protected from HIV and other social harms. In the shorter term, interventions with sex workers, based on evidence reviewed here, implemented at sufficient scale and intensity could contribute to markedly reducing the considerable HIV and social risks that sex workers, their clients and the general population face in sub-Saharan Africa.

## References

[1] Vandepitte J, Lyerla R, Dallabetta G, Crabbe F, Alary M, Buve A (2006). Estimates of the number of female sex workers in different regions of the world. Sex Transm Infect.

[2] Baral S, Beyrer C, Muessig K, Poteat T, Wirtz AL, Decker MR (2012). Burden of HIV among female sex workers in low-income and middle-income countries: a systematic review and meta-analysis. Lancet Infect Dis.

[3] Scorgie F, Chersich MF, Ntaganira I, Gerbase A, Lule F, Lo YR (2012). Socio-demographic characteristics and behavioral risk factors of female sex workers in sub-Saharan Africa: a systematic review. AIDS Behav.

[4] Alary M, Lowndes CM (2004). The central role of clients of female sex workers in the dynamics of heterosexual HIV transmission in sub-Saharan Africa. AIDS.

[5] Chen L, Jha P, Stirling B, Sgaier SK, Daid T, Kaul R (2007). Sexual risk factors for HIV infection in early and advanced HIV epidemics in sub-Saharan Africa: systematic overview of 68 epidemiological studies. PLoS ONE.

[6] UNAIDS (2010). Global report: UNAIDS report on the global AIDS epidemic. UNAIDS/10.11E|JC1958E.

[7] (2009). WHO, UNAIDS, UNICEF. Towards universal access: scaling up priority HIV/AIDS interventions in the health sector: progress report.

[8] Vuylsteke BL, Das A, Dallabetta G, Laga M, Mayer KH, Pizer HF (2009). Preventing HIV among sex workers. HIV prevention: a comprehensive approach.

[9] Morris CN, Ferguson AG (2006). Estimation of the sexual transmission of HIV in Kenya and Uganda on the trans-Africa highway: the continuing role for prevention in high risk groups. Sex Transm Infect.

[10] Rojanapithayakorn W (2006). The 100% condom use programme in Asia. Reprod Health Matters.

[11] Verma R, Shekhar A, Khobragade S, Adhikary R, George B, Ramesh BM (2010). Scale-up and coverage of Avahan: a large-scale HIV-prevention programme among female sex workers and men who have sex with men in four Indian states. Sex Transm Infect.

[12] Boily MC, Pickles M, Vickerman P, Buzdugan R, Isac S, Deering KN (2008). Using mathematical modelling to investigate the plausibility of attributing observed antenatal clinic declines to a female sex worker intervention in Karnataka state, India. AIDS.

[13] Foss AM, Hossain M, Vickerman PT, Watts CH (2007). A systematic review of published evidence on intervention impact on condom use in sub-Saharan Africa and Asia. Sex Transm Infect.

[14] WHO (2005). Toolkit for targeted HIV/AIDS prevention and care in sex work settings.

[15] UNAIDS (2011). UNAIDS terminology guidelines: revised version.

[16] Egger M, Smith GD, Altman DG (1995). Systematic reviews in health care: meta-analysis in context. London: BMJ Publishing Group.

[17] Odek WO, Busza J, Morris CN, Cleland J, Ngugi EN, Ferguson AG (2009). Effects of micro-enterprise services on HIV risk behaviour among female sex workers in Kenya's urban slums. AIDS Behav.

[18] Alary M, Mukenge-Tshibaka L, Bernier F, Geraldo N, Lowndes CM, Meda H (2002). Decline in the prevalence of HIV and sexually transmitted diseases among female sex workers in Cotonou, Benin, 1993–1999. AIDS.

[19] Ngugi EN, Chakkalackal M, Sharma A, Bukusi E, Njoroge B, Kimani J (2007). Sustained changes in sexual behavior by female sex workers after completion of a randomized HIV prevention trial. J Acquir Immune Defic Syndr.

[20] Ghys PD, Diallo MO, Ettiegne-Traore V, Kale K, Tawil O, Carael M (2002). Increase in condom use and decline in HIV and sexually transmitted diseases among female sex workers in Abidjan, Cote d'Ivoire, 1991–1998. AIDS.

[21] Luchters S, Chersich MF, Rinyiru A, Barasa MS, King'ola N, Mandaliya K (2008). Impact of five years of peer-mediated interventions on sexual behavior and sexually transmitted infections among female sex workers in Mombasa, Kenya. BMC Public Health.

[22] Laga M, Alary M, Nzila N, Manoka AT, Tuliza M, Behets F (1994). Condom promotion, sexually transmitted diseases treatment, and declining incidence of HIV-1 infection in female Zairian sex workers. Lancet.

[23] Kaul R, Kimani J, Nagelkerke NJ, Fonck K, Keli F, MacDonald KS (2002). Reduced HIV risk-taking and low HIV incidence after enrollment and risk-reduction counseling in a sexually transmitted disease prevention trial in Nairobi, Kenya. J Acquir Immune Defic Syndr.

[24] Steen R, Vuylsteke B, DeCoito T, Ralepeli S, Fehler G, Conley J (2000). Evidence of declining STD prevalence in a South African mining community following a core-group intervention. Sex Transm Dis.

[25] Thomsen SC, Ombidi W, Toroitich-Ruto C, Wong EL, Tucker HO, Homan R (2006). A prospective study assessing the effects of introducing the female condom in a sex worker population in Mombasa, Kenya. Sex Transm Infect.

[26] Zachariah R, Harries AD, Buhendwa L, Spielman MP, Chantulo A, Bakali E (2003). Acceptability and technical problems of the female condom amongst commercial sex workers in a rural district of Malawi. Trop Doct.

[27] Wechsberg WM, Luseno WK, Lam WK, Parry CD, Morojele NK (2006). Substance use, sexual risk, and violence: HIV prevention intervention with sex workers in Pretoria. AIDS Behav.

[28] Ray S, van De Wijgert J, Mason P, Ndowa F, Maposhere C (2001). Constraints faced by sex workers in use of female and male condoms for safer sex in urban Zimbabwe. J Urban Health.

[29] Williams BG, Taljaard D, Campbell CM, Gouws E, Ndhlovu L, Van Dam J (2003). Changing patterns of knowledge, reported behaviour and sexually transmitted infections in a South African gold mining community. AIDS.

[30] Shahmanesh M, Patel V, Mabey D, Cowan F (2008). Effectiveness of interventions for the prevention of HIV and other sexually transmitted infections in female sex workers in resource poor setting: a systematic review. Trop Med Int Health.

[31] Medley A, Kennedy C, O'Reilly K, Sweat M (2009). Effectiveness of peer education interventions for HIV prevention in developing countries: a systematic review and meta-analysis. AIDS Educ Prev.

[32] Kalanda B (2010). Empowering young sex workers for safer sex in Dowa and Lilongwe Districts of Malawi. Malawi Med J.

[33] Lafort Y, Geelhoed D, Cumba L, Lazaro CD, Delva W, Luchters S (2010). Reproductive health services for populations at high risk of HIV: performance of a night clinic in Tete province, Mozambique. BMC Health Serv Res.

[34] Mukenge-Tshibaka L, Alary M, Geraldo N, Lowndes CM (2005). Incorrect condom use and frequent breakage among female sex workers and their clients. Int J STD AIDS.

[35] Larsen MM, Sartie MT, Musa T, Casey SE, Tommy J, Saldinger M (2004). Changes in HIV/AIDS/STI knowledge, attitudes and practices among commercial sex workers and military forces in Port Loko, Sierra Leone. Disasters.

[36] Yadav G, Saskin R, Ngugi E, Kimani J, Keli F, Fonck K (2005). Associations of sexual risk taking among Kenyan female sex workers after enrollment in an HIV-1 prevention trial. J Acquir Immune Defic Syndr.

[37] McClelland RS, Hassan WM, Lavreys L, Richardson BA, Mandaliya K, Ndinya-Achola J (2006). HIV-1 acquisition and disease progression are associated with decreased high-risk sexual behaviour among Kenyan female sex workers. AIDS.

[38] Wang C, Hawes SE, Gaye A, Sow PS, Ndoye I, Manhart LE (2007). HIV prevalence, previous HIV testing, and condom use with clients and regular partners among Senegalese commercial sex workers. Sex Transm Infect.

[39] Grosskurth H, Mosha F, Todd J, Mwijarubi E, Klokke A, Senkoro K (1995). Impact of improved treatment of sexually transmitted diseases on HIV infection in rural Tanzania: randomised controlled trial. Lancet.

[40] Cohen MS, Chen YQ, McCauley M, Gamble T, Hosseinipour MC, Kumarasamy N (2011). Prevention of HIV-1 infection with early antiretroviral therapy. N Engl J Med.

[41] Steen R, Chersich M, de Vlas SJ (2012). Periodic presumptive treatment of curable sexually transmitted infections among sex workers: recent experience with implementation. Curr Opin Infect Dis.

[42] Steen R, Dallabetta G (2003). Sexually transmitted infection control with sex workers: regular screening and presumptive treatment augment efforts to reduce risk and vulnerability. Reproductive Health Matters.

[43] Steen R, Chersich M, Gerbase A, Neilsen G, Wendland A, Ndowa F (2012). Periodic presumptive treatment of curable sexually transmitted infections among sex workers: a systematic review. AIDS.

[44] Kimani J, Kaul R, Nagelkerke NJ, Luo M, MacDonald KS, Ngugi E (2008). Reduced rates of HIV acquisition during unprotected sex by Kenyan female sex workers predating population declines in HIV prevalence. AIDS.

[45] Mukenge-Tshibaka L, Alary M, Lowndes CM, Van Dyck E, Guedou A, Geraldo N (2002). Syndromic versus laboratory-based diagnosis of cervical infections among female sex workers in Benin: implications of nonattendance for return visits. Sex Transm Dis.

[46] Nagot N, Meda N, Ouangre A, Ouedraogo A, Yaro S, Sombie I (2004). Review of STI and HIV epidemiological data from 1990 to 2001 in urban Burkina Faso: implications for STI and HIV control. Sex Transm Infect.

[47] Ghys PD, Diallo MO, Ettiegne-Traore V, Satten GA, Anoma CK, Maurice C (2001). Effect of interventions to control sexually transmitted disease on the incidence of HIV infection in female sex workers. AIDS.

[48] Meda N, Ndoye I, M'Boup S, Wade A, Ndiaye S, Niang C (1999). Low and stable HIV infection rates in Senegal: natural course of the epidemic or evidence for success of prevention?. AIDS.

[49] Homaifar N, Wasik SZ (2005). Interviews with Senegalese commercial sex trade workers and implications for social programming. Health Care Women Int.

[50] Laurent C, Seck K, Coumba N, Kane T, Samb N, Wade A (2003). Prevalence of HIV and other sexually transmitted infections, and risk behaviours in unregistered sex workers in Dakar, Senegal. AIDS.

[51] Wi T, Ramos ER, Steen R, Esguerra TA, Roces MC, Lim-Quizon MC (2006). STI declines among sex workers and clients following outreach, one time presumptive treatment, and regular screening of sex workers in the Philippines. Sex Transm Infect.

[52] Steen R, Ralepeli S, DeCoito T (2001). Lesedi: services for women at high risk help reduce sexually transmitted infection (STI) prevalence in a South African mining community. FHI/UNAIDS best practices in HIV/AIDS prevention collection.

[53] Cowan FM, Hargrove JW, Langhaug LF, Jaffar S, Mhuriyengwe L, Swarthout TD (2005). The appropriateness of core group interventions using presumptive periodic treatment among rural Zimbabwean women who exchange sex for gifts or money. J Acquir Immune Defic Syndr.

[54] Kaul R, Kimani J, Nagelkerke NJ, Fonck K, Ngugi EN, Keli F (2004). Monthly antibiotic chemoprophylaxis and incidence of sexually transmitted infections and HIV-1 infection in Kenyan sex workers: a randomized controlled trial. JAMA.

[55] Labbé AC, Dzokoto A, Khonde N, Pépin J, Meda H, Asamoah-Adu C (2003). A randomized placebo-controlled trial of routine monthly antibiotics against gonococcal and chlamydial infections among female sex workers in Ghana and Benin: intention-to-treat analysis. 15th Biennial Congress of the International Society for Sexually Transmitted Diseases Research (ISSTDR);.

[56] McClelland RS, Richardson BA, Hassan WM, Chohan V, Lavreys L, Mandaliya K (2008). Improvement of vaginal health for Kenyan women at risk for acquisition of human immunodeficiency virus type 1: results of a randomized trial. J Infect Dis.

[57] Stadler J, Delany S (2006). The ‘healthy brothel’: the context of clinical services for sex workers in Hillbrow, South Africa. Cult Health Sex.

[58] Morin D, Godin G, Alary M, Sawadogo MR, Bernier M, Khonde N (2008). Satisfaction with health services for STIs, HIV, AIDS among a high-risk population in West Africa. AIDS Care.

[59] Vuylsteke B, Traore M, Mah-Bi G, Konan Y, Ghys P, Diarra J (2004). Quality of sexually transmitted infections services for female sex workers in Abidjan, Cote d'Ivoire. Trop Med Int Health.

[60] Reza-Paul S, Beattie T, Syed HU, Venukumar KT, Venugopal MS, Fathima MP (2008). Declines in risk behaviour and sexually transmitted infection prevalence following a community-led HIV preventive intervention among female sex workers in Mysore, India. AIDS.

[61] Wojcicki JM (2002). Commercial sex work or ukuphanda? Sex-for-money exchange in Soweto and Hammanskraal area, South Africa. Cult Med Psychiatry.

[62] Fisher JC, Cook PA, Kapiga SH (2010). Alcohol use before sex and HIV risk: situational characteristics of protected and unprotected encounters among high-risk African women. Sex Transm Dis.

[63] Lewis JJ, Garnett GP, Mhlanga S, Nyamukapa CA, Donnelly CA, Gregson S (2005). Beer halls as a focus for HIV prevention activities in rural Zimbabwe. Sex Transm Dis.

[64] Kalichman SC, Simbayi LC, Vermaak R, Cain D, Smith G, Mthebu J (2008). Randomized trial of a community-based alcohol-related HIV risk-reduction intervention for men and women in Cape Town South Africa. Ann Behav Med.

[65] Chersich MF, Luchters SM, Malonza IM, Mwarogo P, King'ola N, Temmerman M (2007). Heavy episodic drinking among Kenyan female sex workers is associated with unsafe sex, sexual violence and sexually transmitted infections. Int J STD AIDS.

[66] Chersich MF, Rees HV, Scorgie F, Martin G (2009). Enhancing global control of alcohol to reduce unsafe sex and HIV in sub-Saharan Africa. Global Health.

[67] Chisholm D, Rehm J, Van Ommeren M, Monteiro M (2004). Reducing the global burden of hazardous alcohol use: a comparative cost-effectiveness analysis. J Stud Alcohol.

[68] Wechsberg WM, Wu LT, Zule WA, Parry CD, Browne FA, Luseno WK (2009). Substance abuse, treatment needs and access among female sex workers and non-sex workers in Pretoria, South Africa. Subst Abuse Treat Prev Policy.

[69] Elmore-Meegan M, Conroy RM, Agala CB (2004). Sex workers in Kenya, numbers of clients and associated risks: an exploratory survey. Reprod Health Matters.

[70] Ntumbanzondo M, Dubrow R, Niccolai LM, Mwandagalirwa K, Merson MH (2006). Unprotected intercourse for extra money among commercial sex workers in Kinshasa, Democratic Republic of Congo. AIDS Care.

[71] Sutherland EG, Alaii J, Tsui S, Luchters S, Okal J, King'ola N (2011). Contraceptive needs of female sex workers in Kenya - a cross-sectional study. Eur J Contracept Reprod Health Care.

[72] Aklilu M, Messele T, Tsegaye A, Biru T, Mariam DH, van Benthem B (2001). Factors associated with HIV-1 infection among sex workers of Addis Ababa, Ethiopia. AIDS.

[73] Adu-Oppong A, Grimes RM, Ross MW, Risser J, Kessie G (2007). Social and behavioral determinants of consistent condom use among female commercial sex workers in Ghana. AIDS Educ Prev.

[74] Dandona R, Dandona L, Kumar GA, Gutierrez JP, McPherson S, Bertozzi SM (2005). HIV testing among female sex workers in Andhra Pradesh, India. AIDS.

[75] Marks G, Crepaz N, Senterfitt JW, Janssen RS (2005). Meta-analysis of high-risk sexual behavior in persons aware and unaware they are infected with HIV in the United States: implications for HIV prevention programs. J Acquir Immune Defic Syndr.

[76] Denison JA, O'Reilly KR, Schmid GP, Kennedy CE, Sweat MD (2008). HIV voluntary counseling and testing and behavioral risk reduction in developing countries: a meta-analysis, 1990–2005. AIDS Behav.

[77] WHO, UNAIDS. Guidance on provider-initiated HIV testing and counselling in health facilities. http://www.who.int/hiv/pub/guidelines/pitc2007/en/index.html.

[78] Riedner G, Hoffmann O, Rusizoka M, Mmbando D, Maboko L, Grosskurth H (2006). Decline in sexually transmitted infection prevalence and HIV incidence in female barworkers attending prevention and care services in Mbeya Region, Tanzania. AIDS.

[79] Alary M, Gbenafa-Agossa C, Aina G, Ndour M, Labbe AC, Fortin D (2006). Evaluation of a rapid point-of-care test for the detection of gonococcal infection among female sex workers in Benin. Sex Transm Infect.

[80] Vickerman P, Watts C, Peeling RW, Mabey D, Alary M (2006). Modelling the cost effectiveness of rapid point of care diagnostic tests for the control of HIV and other sexually transmitted infections among female sex workers. Sex Transm Infect.

[81] Deceuninck G, Asamoah-Adu C, Khonde N, Pepin J, Frost EH, Deslandes S (2000). Improvement of clinical algorithms for the diagnosis of Neisseria gonorrhoeae and Chlamydia trachomatis by the use of Gram-stained smears among female sex workers in Accra, Ghana. Sex Transm Dis.

[82] Vickerman P, Ndowa F, O'Farrell N, Steen R, Alary M, Delany-Moretlwe S (2010). Using mathematical modelling to estimate the impact of periodic presumptive treatment on the transmission of sexually transmitted infections and HIV among female sex workers. Sex Transm Infect.

[83] WHO (2008). Periodic presumptive treatment for sexually transmitted infections: experience from the field and recommendations for research.

[84] Bosire W, Luchters S, Nel A, Malava E, Malonza I, Temmerman M (2008). A prospective microbicides preparedness study for estimation of HIV-1 incidence among female sex workers in Mombasa, Kenya. In: Microbicides 2008.

[85] Swendeman D, Basu I, Das S, Jana S, Rotheram-Borus MJ (2009). Empowering sex workers in India to reduce vulnerability to HIV and sexually transmitted diseases. Soc Sci Med.

[86] Blankenship KM, Friedman SR, Dworkin S, Mantell JE (2006). Structural interventions: concepts, challenges and opportunities for research. J Urban Health.

[87] Nairne D (2000). “We want the power” findings from focus group discussions in Hillbrow, Johannesburg. Res Sex Work.

[88] Behets FM, Van Damme K, Rasamindrakotroka A, Hobbs M, McClamroch K, Rasolofomanana JR (2005). Socio-demographic and behavioural factors associated with high incidence of sexually transmitted infections in female sex workers in Madagascar following presumptive therapy. Sex Health.

[89] Williams E, Lamson N, Efem S, Weir S, Lamptey P (1992). Implementation of an AIDS prevention program among prostitutes in the Cross River State of Nigeria. AIDS.

[90] Mollet S, Fatoumata (2009). Brothels in Bamako today. Res Sex Work.

[91] (2008). Sex Worker Eduation and Advocacy Taskforce. Sexuality in Africa Magazine.

[92] Richter M (2008). Sex work, reform initiatives and HIV/AIDS in inner-city Johannesburg. Afr J AIDS Res.

[93] Scorgie F, Nakato D, Ogutu AD, Netshivhambe M, Chakuvinga P, Nkomo P “I expect to be abused and I have fear”: sex workers’ experiences of human rights violations and barriers to accessing healthcare in four African countries.

[94] Arnott J, Crago AL (2009). Rights not rescue: a report on female, male, and trans sex workers’ human rights in Botswana, Namibia, and South Africa. Open society initiative for Southern Africa, sexual health and rights project.

[95] Ferguson AG, Morris CN (2007). Mapping transactional sex on the Northern Corridor highway in Kenya. Health Place.

[96] Okal J, Chersich MF, Tsui S, Sutherland E, Temmerman M, Luchters S (2011). Sexual and physical violence against female sex workers in Kenya: a qualitative enquiry. AIDS Care.

[97] Fick N (2007). Well intentioned but misguided? Criminalising sex workers’ clients. Sex Worker Education and Advocacy Taskforce (SWEAT). SA Crime Q.

[98] Pauw I, Brener L (2003). ‘You are just whores – you can't be raped’: barriers to safe sex practices among women street workers in Cape Town. Cult Health Sex.

[99] Gardner JB (2002). Two years of safer sex promotion in escort agencies an massage parlours: a review of an NGO's successes and difficulties.

[100] Steen R, Mogasale V, Wi T, Singh AK, Das A, Daly C (2006). Pursuing scale and quality in STI interventions with sex workers: initial results from Avahan India AIDS Initiative. Sex Transm Infect.

[101] WHO (2000). Regional Office for the Western Pacific: 100% condom use programme in entertainment establishments. Manila, Philippines: WHO.

[102] Kerrigan D, Moreno L, Rosario S, Gomez B, Jerez H, Barrington C (2006). Environmental-structural interventions to reduce HIV/STI risk among female sex workers in the Dominican Republic. Am J Public Health.

[103] Kayembe PK, Mapatano MA, Busangu AF, Nyandwe JK, Musema GM, Kibungu JP (2008). Determinants of consistent condom use among female commercial sex workers in the Democratic Republic of Congo: implications for interventions. Sex Transm Infect.

[104] Morris CN, Morris SR, Ferguson AG (2009). Sexual behavior of female sex workers and access to condoms in Kenya and Uganda on the Trans-Africa highway. AIDS Behav.

[105] Chege MN, Kabiru EW, Mbithi JN, Bwayo JJ (2002). Childcare practices of commercial sex workers. East Afr Med J.

[106] Cornish F, Campbell C (2009). The social conditions for successful peer education: a comparison of two HIV prevention programs run by sex workers in India and South Africa. Am J Community Psychol.

[107] Campbell C (2000). Selling sex in the time of AIDS: the psycho-social context of condom use by sex workers on a Southern African mine. Soc Sci Med.

